# Supplementary material for: Biphasic Npas4 expression promotes inhibitory plasticity and suppression of fear memory consolidation in mice
Source: Mol Psychiatry. 2024 Feb 13;29(7):1929–40. doi: 10.1038/s41380-024-02454-3 (PMC11408256; doi:10.1038/s41380-024-02454-3)
Supplement: Supplementary file 1 — Supplemental Material [file 41380_2024_2454_MOESM1_ESM.pdf]

Supporting Information for
Biphasic Npas4 expression promotes inhibitory plasticity and suppression of fear memory consolidation in mice

Dr. David V.C. Brito[1,2,3,#], Dr. Janina Kupke[1,4#], Rostilav Sokolov[5,6,7], PD Dr. Sidney Cambridge[8], PD Dr. Martin Both[9], Dr. C. Peter Bengtson[1], Dr. Andrei Rozov[6,9,10], and Dr. Ana M.M. Oliveira[1,11*]

[1]Department of Neurobiology, Interdisciplinary Center for Neurosciences (IZN), Heidelberg University, 69120 Heidelberg, Germany

[2]Present address: ABC-RI, Algarve Biomedical Center Research Institute, 8005-139 Faro, Portugal

[3]Present address: Faculdade de Medicina e Ciências Biomédicas, Universidade do Algarve, 8005−139, Faro, Portugal

[4]Present address: Department of Molecular and Cellular Neurobiology, Center for Neurogenomics and Cognitive Research, Amsterdam Neuroscience, Vrije Universiteit Amsterdam, 1081 HV, Netherlands.

[5]Shemyakin-Ovchinnikov Institute of Bioorganic Chemistry, Russian Academy of Sciences, 117997 Moscow, Russia

[6]Federal Center of Brain Research and Neurotechnologies, 117513 Moscow, Russia

[7]Institute of Neuroscience, Lobachevsky State University of Nizhniy Novgorod, Nizhny Novgorod, Russia

[8]Anatomy II, Dr. Senckenberg Anatomy, Goethe-University Frankfurt, 60590 Frankfurt am Main, Germany

[9]Institute of Physiology and Pathophysiology, Medical Faculty, Heidelberg University, 69120 Heidelberg, Germany

[10]OpenLab of Neurobiology, Kazan Federal University, 420008 Kazan, Russia.

[11]Department of Molecular and Cellular Cognition Research, Central Institute of Mental Health, Medical Faculty Mannheim, Heidelberg University, 68159 Mannheim, Germany

[#]DVCB and JK contributed equally to this work. First co-authors are listed alphabetically.

*Ana M.M. Oliveira. Department of Molecular and Cellular Cognition Research, Central Institute of Mental Health, Medical Faculty Mannheim, Heidelberg University, 68159 Mannheim, Germany. Phone: +49(0)62215416510. Email: oliveira@nbio.uni-heidelberg.de, Ana.Oliveira@zi-manheim.de

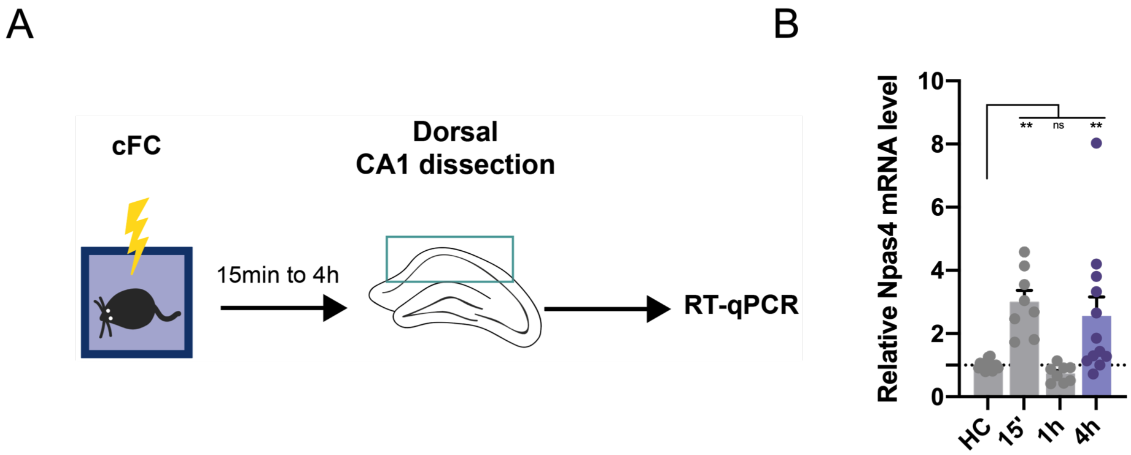

Supplementary Figure 1. Validation of a second high salience contextual paradigm that induces Npas4 biphasic expression. **(A)** Experimental scheme. **(B)** qRT-PCR analysis of CA1 tissue obtained from mice that were left in their home cage (HC) or exposed to high (1x0.7 mA, N=8) salience fear conditioning training and sacrificed 15min, 1h or 4h after (N=8-15). Dots represent individual mice. Data are shown as mean ± standard error of the mean (SEM). One-way ANOVA; Dunnett's; Ns, nonsignificant, ** $p<0.01$.

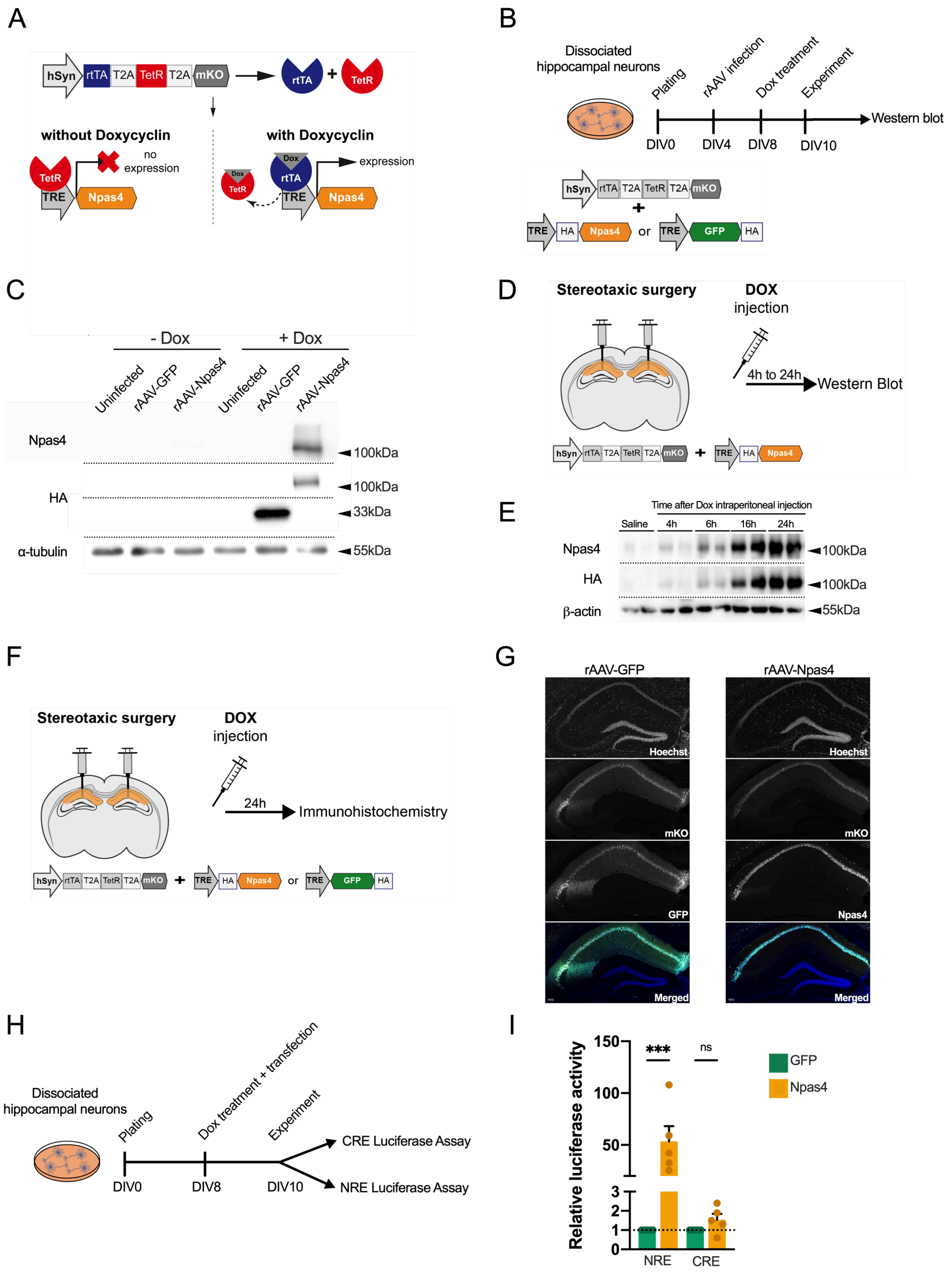

Supplementary Figure 2. Expression and functional characterization of the dual-component TetON-based system. (**A**) Construct scheme. In the absence of doxycycline, the Tet repressor (TetR) is bound to the Tet response element (TRE) promoting active repression of transgene expression (left). In the presence of doxycycline, the TetR loses affinity thus allowing the reverse tetracycline-controlled transactivator (rtTA) to bind to the TRE and initiate transgene expression.

(**B**) Experimental scheme. Primary hippocampal cultures were infected with rAAVs encoding the TetON-based system to express HA-tagged Npas4 or GFP. Doxycycline (Dox) was introduced at DIV8 to induce expression which was evaluated by **(C)** western blot using an antibody against Npas4 or the HA tag of exogenously expressed Npas4 or GFP. **(D)** Experimental scheme. rAAVs were delivered into the CA1 of mice and 3 weeks later mice received intraperitoneal injections of saline or Dox and sacrificed 4h, 6h, 16h or 24h later. **(E)** Expression of exogenous Npas4 was evaluated by western blot. **(F)** Experimental scheme. rAAVs were delivered into the CA1 of mice and 3 weeks later mice received intraperitoneal injections of Dox and sacrificed 24h later. **(G)** Expression of GFP or Npas4 was evaluated by immunofluorescence. Scale bar= 100µm. **(H)** Experimental scheme. Primary hippocampal cultures were transfected with the dual-component TetON-based system and reporter plasmids that expressed luciferase under the control of a minimal promoter and Npas4 responsive elements (NRE) or CREB responsive element (CRE) and were treated with Dox (N=5 independent cell preparations). (**I**) Luciferase assay. Data are shown as mean ± standard error of the mean (SEM). Dots represent individual cell culture preparations. One-way ANOVA; Dunnett's; Ns, nonsignificant, *** $p<0.001$.
